# The use of biocomposite vented anchors for arthroscopic remplissage allows for better bony ingrowth than peek vented anchors: A volumetric CT study of 85 anchors

**DOI:** 10.1002/jeo2.70605

**Published:** 2025-12-28

**Authors:** Miguel Angel Ruiz Ibán, Rosa Vega, Raquel Ruiz Díaz, Cristina Delgado, Umile Giuseppe Longo, Jorge Diaz Heredia

**Affiliations:** ^1^ Shoulder and Elbow Unit, Orthopaedic Surgery and Trauma Service Hospital Universitario Ramón y Cajal Madrid Spain; ^2^ Departamento de Cirugía, Ciencias Sanitarias Y Medicosociales, Área de Traumatología y Ortopedia Universidad de Alcalá Alcalá de Henares Madrid Spain; ^3^ Shoulder and Elbow Reconstructive Surgery UnitDepartment of Orthopaedic Surgery and Traumatology, Hospital Universitario Fundación Jiménez Diaz Avenida Reyes Catolicos 2 Madrid Spain; ^4^ Fondazione Policlinico Universitario Campus Bio‐Medico Roma Italy; ^5^ Department of Medicine and Surgery, Research Unit of Orthopaedic and Trauma Surgery Università Campus Bio‐Medico di Roma Roma Italy

**Keywords:** arthroscopy, biocomposite, bony ingrowth, humeral cyst, osteolysis, PEEK, remplissage

## Abstract

**Purpose:**

To evaluate whether the use of polyetheretherketone (PEEK) or biocomposite vented anchors has any impact on bony ingrowth inside the anchor or implant‐related osteolysis after implantation during remplissage for Hill‐Sachs defects (HSD) in patients with shoulder instability.

**Methods:**

Prospective cohort study with a minimum of 24 months follow‐up (mean follow‐up 3.11[SD = 0.67] years). Forty‐nine subjects (43 males and 6 females; mean age 27.6[SD:9.22] years) with HSD undergoing remplissage were evaluated with a CT performed at a mean of 14.7[4,4] months after surgery. The procedures were performed in two cohorts: in the first, 44 anchors (4.5 mm Healicoil PEEK) were used in 26 subjects. In the second, 41 anchors (4.75 mm Healicoil Regenesorb biocomposite) were used in 21 subjects. A computed tomography evaluation of the volume of the bone defects and the degree of bony ingrowth was made.

**Results:**

Preoperatively both cohorts were homogenous. The biocomposite anchors showed better bony ingrowth than the PEEK anchors (*p* = 0.0014): full bony ingrowth in 18/41(44%) in biocomposite versus 13/44(30%) in PEEK; clear ossification with a thin lucent rim in 15/41(37%) biocomposite versus 7/44(30%) PEEK; discontinuous ossification in 6/41(15%) biocomposite versus 7/44(16%) PEEK; and no ossification in 2/41(5%) biocomposite versus 17/44(39%) PEEK. The biocomposite anchors showed smaller bone defects than the PEEK anchors (*p* = 0.0217): no bone defect in 18/41(44%) biocomposite versus 13/44(30%) PEEK, partial bone defects in 20/41(49%) biocomposite versus 17/44(39%) PEEK; and bone defects larger than the insertion hole in 2/41(5%) biocomposite versus 13/44(30%) PEEK. One anchor in each group caused a bone defect larger than twice the size of the hole (2%). At the latest follow‐up no differences in clinical outcomes between the groups were found.

**Conclusion:**

Biocomposite vented anchors for remplissage favours bony ingrowth and lower the incidence of osteolysis and defect formation when compared to PEEK anchors with similar clinical outcomes.

**Level of Evidence:**

Level II, prospective cohort study.

**Abbreviations**
CS: calcium sulphate
CT: computed tomography
HSD: Hill‐Sachs defects
PEEK: polyetheretherketone
PLGA: poly‐l‐lactic co‐glycolic acid
β‐TCP: β‐tricalcium phosphate

## INTRODUCTION

Since its introduction in the 1990s, shoulder arthroscopy has evolved into the preferred technique for managing rotator cuff and instability in the shoulder. This transition has been driven, in part, by advancements in arthroscopic instrumentation and the development of diverse anchor types, including the transition from metallic solid anchors to absorbable polylactic acid anchors.

Traditional anchors have some issues, with metallic anchors being associated with chondral damage and absorbable anchors prone to cyst formation [9, 16]. Recently, the use of non‐absorbable polyetheretherketone (PEEK) anchors and biocomposite anchors, composed of materials that enable gradual resorption, has become increasingly widespread [12, 29]. These advanced materials allow for innovative anchor designs, such as open‐architecture (‘vented’) configurations, which have the potential to improve healing rates [10, 13] and mitigate the risk of cyst formation [13, 34].

The Hill‐Sachs defect (HSD) is the most common humeral lesion present in anterior shoulder instability patients and plays an important role in the surgical outcomes of surgery [4, 20, 22, 23, 25, 30]. The remplissage technique is often used to manage these defects [24, 28] as it lowers the recurrence rate when used to augment an arthroscopic Bankart repair [31, 37]. It can be performed with different anchors: metallic anchors [28], cuff or labral repair resorbable anchors [11, 18] and all‐suture anchors [7, 33]. Anchor‐related cyst formation in the humerus in this procedure has an incidence between 2% and 18% [33, 34]. Although the importance of humeral cyst formation in subjects with shoulder instability has not been widely studied, a decrease in the humeral bone stock could increase the size of the bone defect and thus increase the failure rate after arthroscopic soft tissue repair [3].

The objective of this study was to determine whether the use of PEEK or biocomposite resorbable open‐architecture anchors during arthroscopic remplissage influences the extent of bony ingrowth within the anchors, the occurrence of anchor‐related osteolysis, or clinical outcomes. The null hypothesis is that no significant differences would be observed between the two materials.

## MATERIALS AND METHODS

The study was approved by the Institutional review Board of Hospital Universitario Ramón y Cajal (approval number 126‐18). Patients gave written informed consent, after receiving detailed oral and written information about the study, for participation in the study.

This was a prospective comparative two‐cohort study. A total of 49 subjects were included in the study simultaneously in two different centres and followed for a minimum of 24 months. Cohort allocation was made according to the centre in which the procedure was performed. Throughout the course of the study, no modifications were made to the study methodology.

### Participants

The inclusion criteria for participants were: (1) age 18 years or older, (2) recurrent anterior shoulder instability requiring a Bankart repair, (3) undergoing arthroscopic remplissage using open‐architecture PEEK or biocomposite anchors, and (4) the ability to understand the study design and provide informed consent to participate.

The exclusion criteria for participants were: (1) a history of previous surgical intervention on the ipsilateral shoulder, or (2) the use of additional anchors (either absorbable or metallic) in the humerus during the procedure.

### Interventions

All procedures were performed at two institutions by the same senior author (MRI) between 2018 and 2020. Subjects scheduled for arthroscopic Bankart repair with an associated remplissage procedure were assessed for eligibility. Participants who met all the inclusion criteria and none of the exclusion criteria were invited by the surgeon to participate in the study. After obtaining informed consent, participants were enroled, a non‐contrast computed tomography (CT) scan of the shoulder was performed at the one‐year follow‐up, and clinical assessments was conducted at a minimum of two‐year follow‐up.

**Figure 1:**
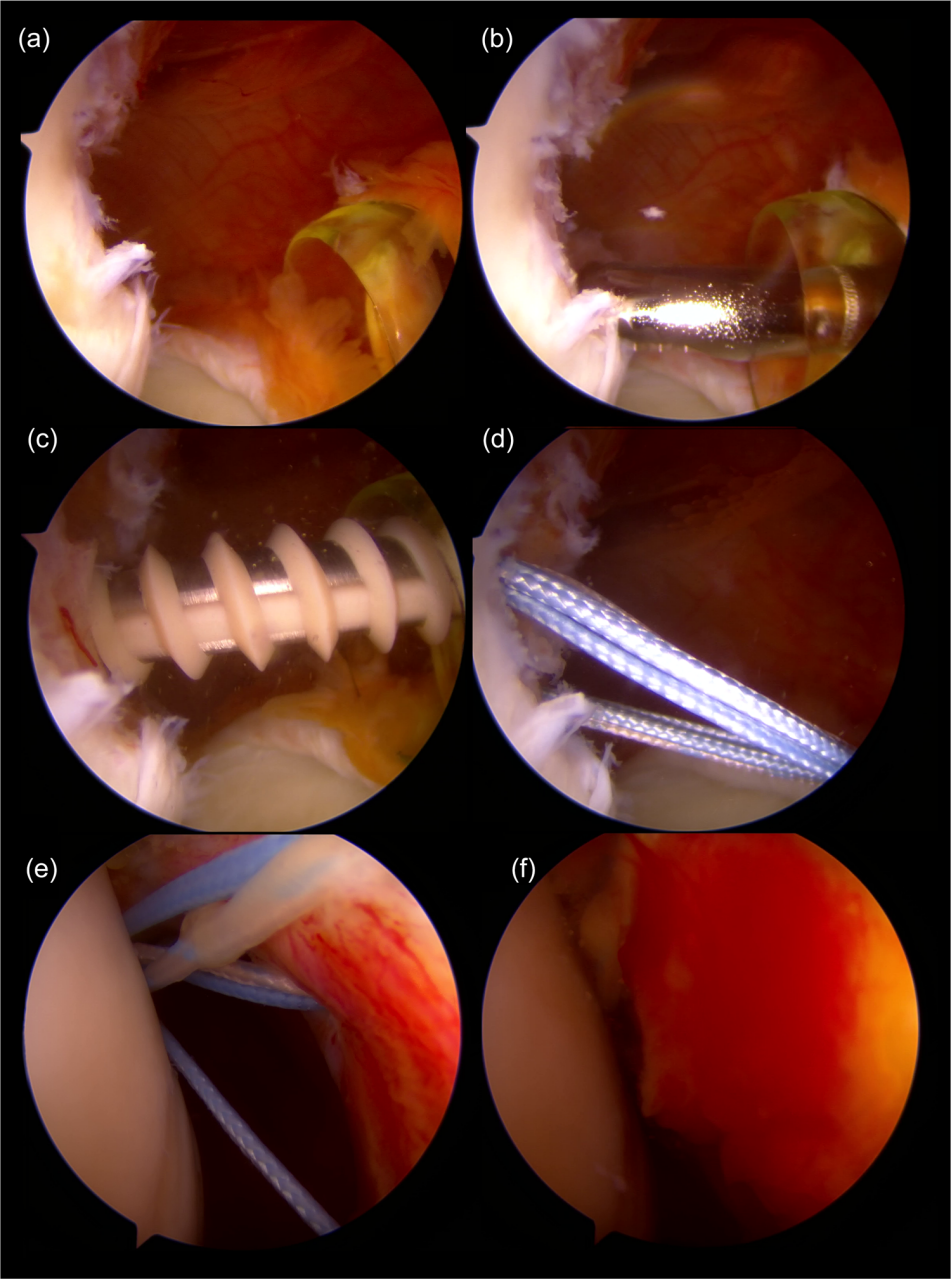
Arthroscopic remplissage technique in a right shoulder. All images taken from a anterosuperior portal aiming at the posterior part of the glenohumeral joint. The Hill‐Sachs lesion is identified (a) and the surface of the humeral defect is gently debrided with a soft tissue motorised shaver (b). One anchor, in this case a 4.5 mm Healicoil polyetheretherketone (PEEK) anchor, is placed at the medial edge of the bone defect though the posterior portal (c and d) and, through an auxiliary posterolateral portal, a suture‐passing device is used to pass the sutures through the capsule and the infraspinatus tendon (e). To finish the sutures are tied in the subacromial space, bringing the capsule and tendon to the bone defect (f).

The arthroscopic remplissage procedure (Figure 1) was indicated for patients undergoing an arthroscopic Bankart procedure which presented, on CT or arthroscopic assessment, with a HSD larger than 15 mm. The procedure was performed under an interscalene nerve block combined with general anaesthesia. Following a comprehensive diagnostic glenohumeral arthroscopy, the HSD was identified, and the necessity for a remplissage was confirmed. The surface of the humeral defect was gently debrided using a motorised soft tissue shaver. The number of anchors used (either one or two) was determined based on the craniocaudal size of the HSD; defects larger than 2 cm craniocaudally were treated with two anchors. The anchors were inserted at the medial edge of the bone defect, close to the cartilage, and sutures were passed through the capsule and infraspinatus tendon using a suture‐passing device via an accesory posterolateral portal. An anterior Bankart repair was performed concurrently using three to five 1.7 mm single‐loaded Suturefix (Smith & Nephew, Andover, USA) anchors. Finally, the remplissage was completed by blindly tying all suture limbs over the infraspinatus through the accessory posterolateral portal.

After surgery, the subjects were immobilised in a sling for three weeks, although gentle passive range of motion (ROM) exercises were allowed. Full passive ROM exercises were initiated on the fourth week, and active assisted ROM exercises were started in the fifth week. During that time the patients and physical therapist were instructed to avoid external rotation beyond 0° until the seventh week. Strength training was started 10 weeks after surgery and the subjects were authorised to go back to sport when 90% of the contralateral ROM in the four planes and 90% of strength was regained.

**Figure 2:**
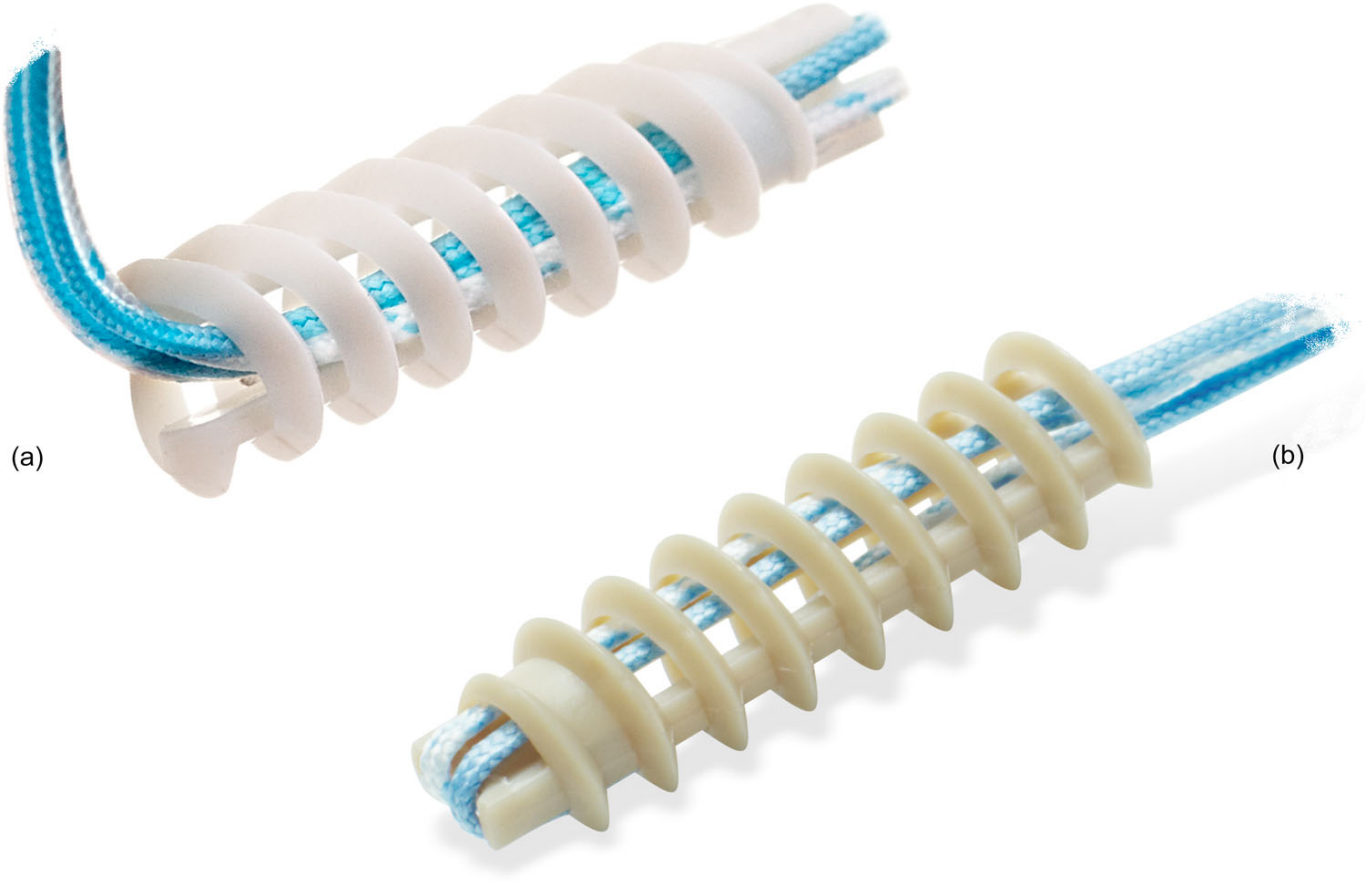
The anchors used in the study. (a) The 4.75 Regenesorb Healicoil (Smith&Nephew, Andover. USA) made of Regenesorb material, a co‐polymer of 65% PLGA (poly‐l‐lactic co‐glycolic acid), 15% β‐tricalcium phosphate, and 20% calcium sulphate. (b) The 4.5 PEEK (polyetheretherketone) Healicoil (Smith&Nephew, Andover. USA) made of polyetheretherketone.

The anchor composition, either PEEK or biocomposite, was selected depending on the institution in which the procedure was performed: in one institution (Hospital Universitario Ramon y Cajal, Madrid, Spain) only 4.5 PEEK Healicoil (Smith&Nephew, Andover. USA) anchors were used; in the other institution (Hospital Universitario HM Sanchinarro, Madrid, Spain) only 4.75 Regenesorb Healicoil (Smith&Nephew, Andover. USA) anchors were used (Figure 2). Both cohorts were recruited simultaneously by the surgeon in each institution. The length of the anchors was 18.5 mm. The Regenesorb material is a co‐polymer consisting of 65% PLGA (poly‐l‐lactic co‐glycolic acid), 15% β‐tricalcium phosphate (β‐TCP), and 20% calcium sulphate (CS). The three components, PLGA, β‐TCP, and CS, have different intraosseous resorption characteristics: PLGA, a co‐polymer made of polylactic acid (PLLA) and polyglycolic acid (PGA) in a ratio of 85:15, has a resorption time of 24 months; [1] the other two components have reported shorter resorption times (18 months for β‐TCP, and 4–12 weeks for CS) [19, 36].

### Outcomes

As a preliminary investigation, to assess the volume of the insertion hole caused during anchor insertion, six 4.5 mm Healicoil PEEK and six 4.75 mm Healicoil biocompositeRegenesorb (Smith&Nephew, Boston, USA) anchors were placed inside a Solid Foam Block (Sawbones, Washington, USA) with a density of 20 PFC, which is roughly equivalent to hard cancellous bone. All anchors were placed sequentially, one cm apart, using the different specific awls provided by the manufacturer to prepare the insertion place. Then the block underwent a high resolution (0.25 mm slice) non‐contrast CT scan. The volume of the resulting insertion holes was measured in the images by an orthopaedic surgeon (CD) with extensive experience using the OsiriX DICOM viewer for Mac software (version 11, Pixmeo, Geneva, Switzerland) as defined by Ruiz Iban et al. [34]. To ensure accuracy, all measurements were made thrice and the median of the measurements for each anchor type was used as reference for insertion hole size. The surgeon (CD) making the measurements was blinded to the nature of the anchors used.

The surgical records of each participant were reviewed, and the number and position in the humerus of each anchor were noted. The CT DICOM data were imported to the OsiriX DICOM viewer after anonymization. Any bone defects that corresponded to the anchors placed in the humerus were identified in the axial slices of the humerus and correlated with the surgery data. The density of the content of the anchor track was compared to adjacent cancellous bone using Hounsfield units to define whether it was soft tissue or cancellous bone. The volumes of the remaining bone defects were calculated and compared to the insertion hole volume. Each bone defect was measured thrice, and the mean was used for calculations. This measurement method has showed a high intraobserver and interobserver reproducibility (intraclass correlation coefficient > 0.9) [33].

The primary outcome was whether an anchor had caused a relevant bone defect in the humeral head. Every anchor placed was located in the CT and the ingrowth pattern was evaluated using the Barber classification [2] that classifies bony ingrowth in four types: type 1, in which little or no ossification is found within the anchor tract, which is filled with material having a density consistent with soft tissue; type 2 in which some ossification, discontinuous or with a wide lucent rim is present. with density still consistent with soft tissue, type 3 in which ossification is present with a thin lucent rim and the density is consistent with cancellous bone; and type 4, with good ossification, vague tract border and density consistent with cancellous bone.

Additionally, each of the defects were classified according to its volume, as suggested by Ruiz Ibán et al. [32, 33, 34], into one of four groups: (1) full ingrowth of the bone defect with all the anchor filled with cancellous bone (this group corresponded to Barber's 4 ingrowth type); (2) partial ingrowth of the anchor with a visible defect smaller in size than the insertion hole, suggesting partial bony ingrowth into the anchor; (3) hole enlargement without relevant bony ingrowth and a bone defect that is larger than the insertion hole but smaller than twice that volume; and (4) cystic lesions defined as bone defects larger than twice that volume (200%) of that produced with the insertion hole. This measurement was adjusted according to the volume the defect caused by each anchor type during insertion in the foam blocks.

At the minimum 24‐month follow‐up, clinical evaluation included a physical examination and the assessment of Constant–Murley and Rowe scores using validated Spanish questionnaires. Additionally, any complications, recurrence of instability, or persistent apprehension were documented.

### Statistical analysis

All continuous variables were tested for normality using the Kolmogorov–Smirnov test. Chi‐squared test was used to compare dichotomous and qualitative variables. Student's t‐test was used to compare quantitative variables. A logistic regression analysis was performed to assess the possible effect of sex, age, number of anchors, and time to CT in the size of the bone defects. The statistical threshold for significance was established at *p* < 0.05.

The sample size was calculated before the start of the study. The estimated incidence of bony ingrowth at the anchor site used for sample size calculation was 50%, this number was obtained from the available literature [2, 34]. Assuming that the use of biocomposite anchors increased the degree of bony ingrowth by 50%, for a 0.75 power and a 95% confidence interval, an estimated 40 anchors per group should be evaluated. As one or two anchors are used in each case for remplissage, the recruitment target was established at *n* = 27 per group. Estimating a 10% loss of follow‐up a final sample size of 30 subjects was selected for each group.

## RESULTS

Sixty subjects (30 from each institution) met the inclusion criteria, consented to participate in the study, and signed the informed consent form. Of these, 49 (81.6%)—27 from one institution and 22 from the other—completed a 1‐year follow‐up CT evaluation and a minimum 2‐year clinical follow‐up and were subsequently included in the data analysis.

The clinical and epidemiological characteristics of the two cohorts of subjects are reported in Table 1. Both samples were homogeneous according to preoperative data. During the surgical procedure, a HSD was identified in all the patients, and a remplissage procedure was performed for every lesion. Among the 49 subjects, a total of 85 anchors were used for remplissage, comprising 44 PEEK anchors and 41 biocomposite anchors. A single anchor was used in 11 cases, while two anchors were employed in the remaining 37 subjects. All 49 participants underwent a CT scan at a mean of 14.7 (standard deviation: 4.4 months) months post‐surgery.

**Table 1:** Baseline demographic and clinical characteristics for each group.

	Total (*n* = 49)	PEEK group (*n* = 27)	Biocomposite group (*N* = 22)	*p* value
Demographic data:				
Age (years)	27.6 ± 9.22	27.7 ± 5.0	27.4 ± 8.7	NS
Sex (male:female)	43:6	24:3	19:3	NS
Instability characteristics				
Side (Right:Left)	25:24	12:15	13:10	NS
Dominant side affected	27 (55.1%)	15 (55.5%)	12 (54.5%)	NS
Number of previous dislocations	6.2 ± 5.8	6.4 ± 5.0	6.0 ± 6.8	NS
Glenoid defect:				
Size (mm)	7.4 ± 3.8	7.5 ± 3.8	7.3 ± 3.7	NS
0%–5%	13 (26.5%)	8 (29.6%)	5 (22.7%)	
5%–10%	24 (49.0%)	12 (44.4%)	12 (54.5%)	NS
10%–15%	12 (24.5%)	7 (25.9%)	5 (22.7%)	
Hill‐Sachs defect: (mm)				
Cranio‐caudal	20.2 ± 3.7	20.3 ± 3.9	20.0 ± 3.6	NS
Medio‐lateral	14.8 ± 6.0	14.9 ± 6.0	14.7 ± 6.1	NS
Depth	10.1 ± 3.9	10.5 ± 3.8	9.5 ± 4.0	NS
Constant‐Murley score	82.1 ± 8,11	83.1 ± 7.97	81.0 ± 8.47	NS
Rowe score	29.1 ± 8.93	28.5 ± 8.85	29.9 ± 9.19	NS

*Note*: The values are given as mean ± standard deviation for quantitative variables and number (percentage of total) for quantitative variables. There were no significant differences between the two cohorts.

Abbreviations: BMI: body mass index; PEEK, polyetheretherketone.

The median volume of the insertion holes caused during anchor insertion in the foam blocks was 285 mm^3^ for the PEEK anchors and 334mm^3^ for the biocomposite anchors.

**Figure 3:**
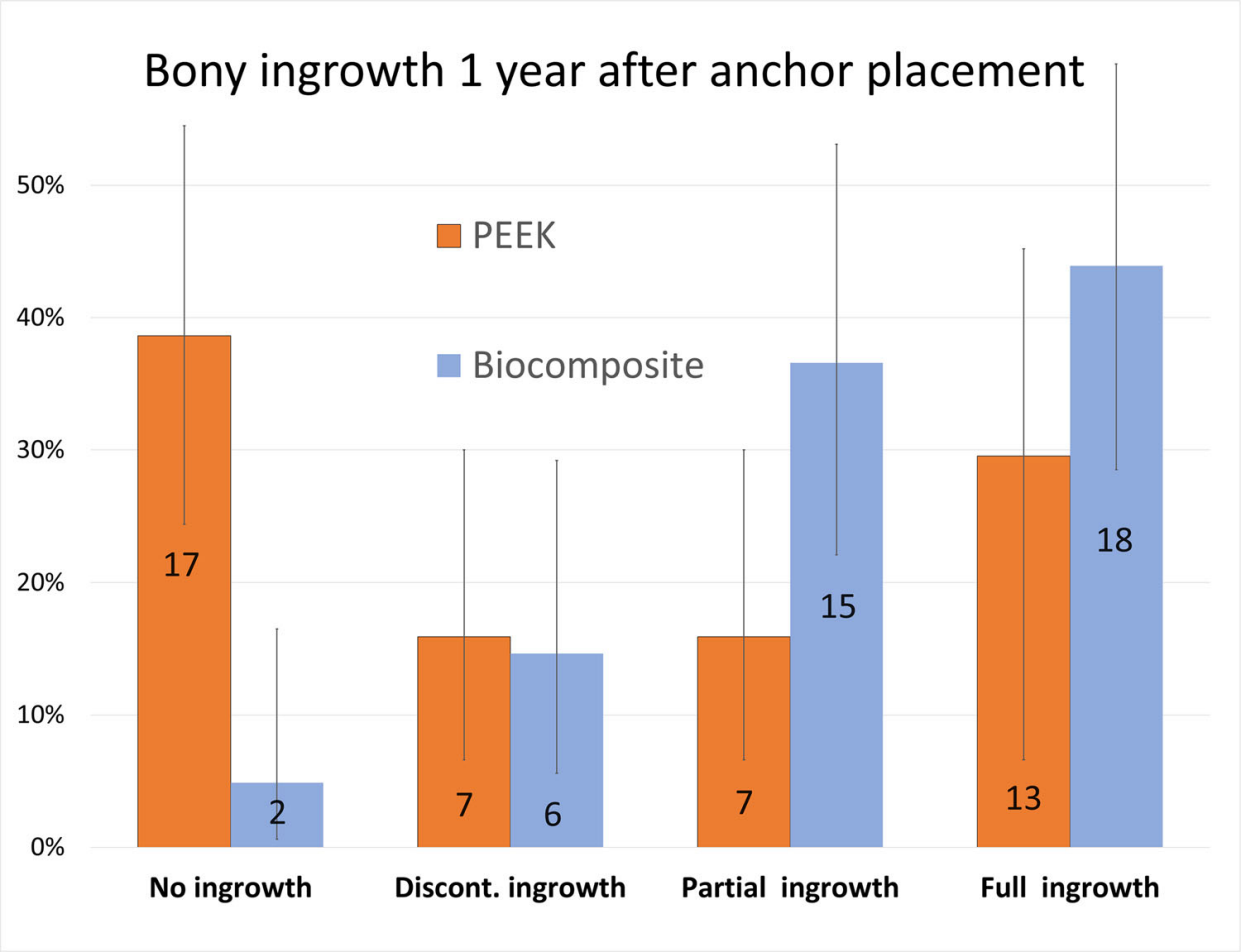
Computed tomography assessment of the bony ingrowth inside the 85 humeral bone defects caused by the vented polyetheretherketone (PEEK) and Biocomposite anchors at 1‐year follow‐up, evaluated according to Barber et al. [2]. The diagram shows the relative incidence for each type, including the 95% confidence interval. The numbers overlaid on the graph represent the absolute numbers. There were significant differences, with more bony ingrowth in the Biocomposite anchors (*χ* ^2^ = 15.5; *p* = 0.0014).

**Figure 4:**
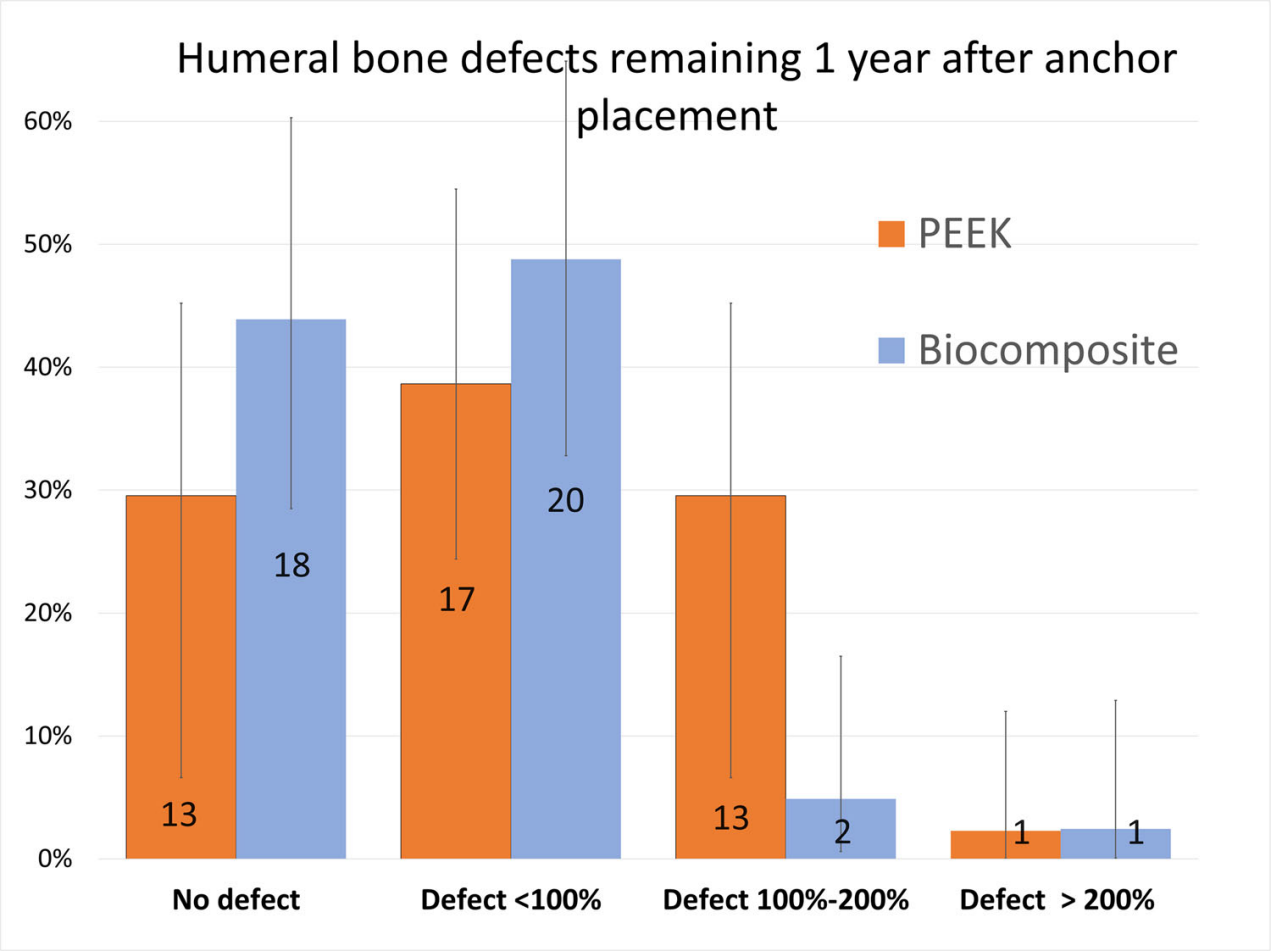
Computed tomography assessment of the size of the 85 remaining humeral bone defect caused by the vented polyetheretherketone (PEEK) and Biocomposite anchors at 1‐year follow‐up, evaluated according to Ruiz Iban et al. [28]. The diagram shows the relative incidence for each type, including the 95% confidence interval. The numbers overlaid on the graph represent the absolute numbers. There were significant differences, with smaller defects in the Biocomposite anchors ( *χ* ^2^ = 9.02; *p* = 0.0217).

The anchor insertion zones of the 85 anchors could be easily identified in all 49 CT scans. There were significant differences in the degree of bony ingrowth between the two groups, with the biocomposite anchors having better bony ingrowth than the PEEK anchors (*χ* ^2^ = 15.5; *p* = 0.0014) (Figure 3). There were also significant differences in the remaining defect volume between the two groups, with the biocomposite anchors having smaller defect volumes than the PEEK anchors (*χ* ^2^ = 9.02; *p* = 0.0217) (Figure 4).

There was no correlation between the number of anchors used or its position in an individual subject and the size of the bone defects. Multivariate regression analysis did not show a statistically significant association between sex, age, number of humeral anchors, or time to CT with the degree of bone ingrowth or size of the remaining defects (NS).

Clinical evaluation was performed at a mean of 3.11 ± 0.67 years after the surgical procedure. There were significant improvements in both the Constant‐Murley Score (from 82.1 ± 8.11 preoperatively to 94.7 ± 2.62 postoperatively, *p* < 0.0001) and the Rowe Score (from 29.1 ± 8.93 preoperatively to 94.5 ± 9.03 postoperatively, *p* < 0.0001) after the procedure but there were no significant differences between both groups in either the Constant‐Murley Score (94.4 ± 2.41 in the PEEK group vs. 95.0 ± 2.89 in the Biocomposite group, NS) or the Rowe Score (95.7 ± 4.32 in the PEEK group vs. 92.9 ± 12.6 in the Biocomposite group, NS). No early postoperative complications developed. One patient (from the Biocomposite Group) suffered a recurrence 15 months after surgery and was revised with a Latarjet procedure and another two (one from each group) had residual apprehension but did not require further surgical procedures (differences not significant).

## DISCUSSION

The most important finding of this study is that the use of open architecture biocomposite anchors during a remplissage procedure allows for better bony ingrowth inside the anchor and causes less residual bony defects than PEEK anchors of similar size and architecture when assessed in CT scans performed one year after surgery, without relevant clinical differences.

There is controversy regarding the optimal material composition for anchors used in arthroscopic shoulder surgery. Metallic anchors, initially developed for their excellent mechanical properties, have fallen out of favour due to their potential to cause significant chondral damage if left protruding or if migration occurs after surgery [26, 29]. Absorbable anchors were later developed and used in various combinations of polylactic acid and polyglycolic acid, but early reabsorption of the anchors with a combination of giant cell reaction and synovitis was associated with osteolysis and bone cyst formation in 10%–30% of cases [9, 16, 27]. All‐suture anchors are used both in rotator cuff repair and instability procedures with success [21, 32, 33] but seem to be associated with significant cyst formation in up to 40% of cases in rotator cuff repair [14]. Lately, both inert non‐absorbable plastic PEEK anchors and absorbable anchors made of a combination of materials that allow for a more gradual resorption (biocomposite anchors) have been introduced to the market as the material composition allows for innovative anchor shapes. In particular ‘Vented’ or ‘open‐architecture’ architectures, with wide holes in the anchor design that allow communication between the soft tissue repair and the cell‐and‐growth factor‐rich cancellous, bone deep in the humeral head, are popular as they might improve cuff healing rates [10, 13] and could avoid cyst formation, as they allow for bone ingrowth inside the anchor [13].

The excellent biological properties of biocomposite anchors found in this study, with full reabsorption and anecdotal cyst formation, is in line with what is found in the literature: Vonhoegen et al. analysed 82 biocomposite (PLGA/ß‐TCP)/CS) anchors used for rotator cuff repair with MRI at one year follow‐up and found results similar to those presented here: full degradation in 50% of anchors and osteolysis in only 2.4% of cases [35]. Barber et al. examined 28 of these anchors with CT scanning at 3‐year follow‐up, and found a bony ingrowth rate of 50%, similar to the 80% rate found in this study [2]. To finish, Kim et al. found cyst formation in 12% of cases when biocomposite anchors were used for rotator cuff repair, but the anchors used were non‐vented [15].

Direct comparisons between biocomposite and other anchors have been reported in the literature too: Kim et al. compared open architecture PEEK anchors with non‐vented biocomposite anchors and did not find clinical or radiological (in CT scans at 6 months postoperative) differences [13]. Kim et al. compared non‐vented biocomposite, all‐suture and PEEK anchors in 73 subjects undergoing rotator cuff repair and did not find clinical or radiological (in MRI) differences between groups [17]. This is in contrast with our results as we did find a better ingrowth profile using biocomposite anchors, this is probably due to the use of vented anchors, which seem to have an advantage over non‐vented traditional anchors [5]. Thus, our results suggest that there is an added advantage of using a vented biocomposite anchor design, as cyst formation is rare (<10%) and bone ingrowth inside the anchor develops in 80% of anchors.

The relevance of the development of osteolytic lesions in the humerus after a remplissage is unclear, as cysts formation has not been directly associated with increased recurrence rates. Despite of this, if a recurrence occurs over a humeral bone weakened by cyst formation, the Hill‐Sachs lesion might increase in size and depth, complicating further procedures [4, 30]. Nevertheless, the remplissage procedure is a good model to obtain data that could be applied to the typical setting in which these anchors are used, rotator cuff repair, in which these osteolytic lesions might have an increased impact in case of revision surgery.

The clinical outcomes of both cohorts were good and in line with found by many different authors [8, 31] and no clinical differences were found between cohorts. This study has a relatively short clinical follow‐up, with recurrence of instability symptoms in only 6% of cases at three‐year follow‐up, in line with previous literature analysing the outcomes of associated Bankart‐remplissage procedures [8].

This study has some limitations. The first is its limited sample size, which do not allow for precise assessment of relatively rare complications such as cyst formation. Anyway, the study was powered primarily to assess bony ingrowth and succeeded finding relevant differences between groups. Also, the natural history of bony ingrowth or cyst formation could not be properly assessed as the radiological assessment was performed only once, and cysts associated with biocomposite anchors have been shown to regress with time [6]. Ideally serial CT scans of the subjects would allow for a more detailed picture, but the subjects would suffer from unacceptable amounts of radiation. To finish, a randomised controlled trial would add robustness to our data, but the cohorts in our study were homogeneous and other variables such as surgical technique and anchor shape where well controlled: exactly the same shape of anchor was used in both anchors studied and all procedures were performed by the same surgeon.

## CONCLUSIONS

Biocomposite vented anchors for remplissage favour bony ingrowth and lower the incidence of bone cyst formation when compared to PEEK anchors without affecting clinical outcomes.

## AUTHOR CONTRIBUTIONS

**Miguel Angel Ruiz Ibán**: conceived the study, wrote the first drawn of the manuscript. **Rosa Vega** and **Cristina Delgado**: data collection. **Umile Giuseppe Longo, Raquel Ruiz Díaz**, and **Jorge Diaz Heredia**: provided resources and contributed to the final manuscript.

## CONFLICT OF INTEREST STATEMENT

The authors declare that their immediate families, or any research foundation with which they are affiliated did not receive any financial payments or other benefits from any commercial entity related to the subject of this article.

## ETHICS STATEMENT

This study was approved by the Institutional review Board of Hospital Universitario Ramón y Cajal (approval number 126‐18; Appendix 1).

## Data Availability

Data available on request from the authors.
